# Transformation of health care and the new model of care in Saudi Arabia: Kingdom’s Vision 2030

**DOI:** 10.25122/jml-2021-0070

**Published:** 2021

**Authors:** Sharfuddin Chowdhury, Dennis Mok, Luke Leenen

**Affiliations:** 1. Trauma Center, King Saud Medical City, Riyadh, Saudi Arabia; 2. Medical Management Consulting, Birkdale, Queensland, Australia; 3. Department of Trauma, University Medical Center Utrecht, Utrecht, The Netherlands

**Keywords:** Saudi Arabia, global health, organizational models, health policy, delivery of health care, KPI – Key Performance Indicator, VRP – Vision Realization Program, NTP – National Transformation Program, MOC – Model of Care, MOH – Ministry of Health, ACO – Accountable Care Organization, SOC – Systems of Care, VRO – Vision Realization Office, CDG – Care Design Group, CDC – Center for Disease Control, KSA – Kingdom of Saudi Arabia, KSMC – King Saud Medical City, Riyadh, KFMC – King Fahad Medical City, Riyadh, KKESH – King Khalid Eye Specialist Hospital, Riyadh, KAMC – King Abdullah Medical City, Mecca, KFSH-D – King Fahad Specialist Hospital, Dammam, CBAHI – The Saudi Central Board for Accreditation of Healthcare Institutions

## Abstract

The Kingdom of Saudi Arabia espoused “Vision 2030” as a strategy for economic development and national growth. The vision demonstrated the Kingdom’s objectives to become a pioneer nation globally by achieving three main goals: a vibrant society, a thriving economy, and an ambitious nation. To fulfill this, the Kingdom launched a national transformation program (NTP) as outlined in “vision 2030” in June 2016. The health care transformation is one of the eight themes of the NTP’s. The history of health care facilities in the Kingdom is almost a century. Although the Kingdom has made notable progress in improving its population’s health over recent decades, it needs to modernize the health care system to reach the “vision 2030” goal. This article aims to describe the new Model of Care (MOC) according to the recent Saudi health care transformation under the Kingdom’s vision 2030. The MOC concept started with understanding the current state and collecting learnings. It is based on the six systems of care (SOC)- keeping well, planned procedure, women & children, urgent problems, chronic conditions, and the last phase of life. The SOC is cut across different “service layers” to support people’s stay well and efficiently get them healthy again when they need care. The new MOC describes a total of forty-two interventions, of which twenty-seven split across the six SOC and the rest fifteen cut-across the multiple SOC. Implementation of all MOC interventions will streamline the Saudi health care system to embrace the Kingdom’s “vision 2030”.

## INTRODUCTION

The Kingdom of Saudi Arabia is the largest country in the Arabian Peninsula, with a landmass of 2250,000 square kilometers and an estimated 33.4 million population [1, 2]. It is considered an energy superpower and one of the world’s top twenty economies [3]. The Kingdom espoused “Vision 2030” as a strategy for economic development and national growth. The vision demonstrated the Kingdom’s objectives to become a pioneer nation globally by achieving three main goals: a vibrant society, a thriving economy, and an ambitious nation. In April 2016, the Kingdom launched its “Vision 2030”, comprised of 96 strategic objectives, governed by several Key Performance Indicators (KPIs). A few initiatives, known as vision realization programs (VRPs), were developed in this regard and under the different governmental, private, and non-profit organizations’ implementation processes to achieve that goal. A practical and integrated governance model was set up by the Council of Economic and Development Affairs to translate “Vision 2030” into multiple VRPs working parallel to achieve the strategic objectives & realize the vision [4, 5]. In June 2016, the National Transformation Program (NTP) was launched as a VRP involving 24 government agencies to build the capacity and capabilities required to achieve the ambitious goals of “Vision 2030” [4, 5].

**Figure 1:**
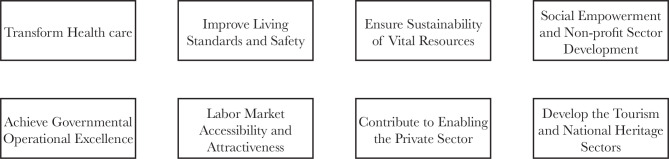
The Themes of the National Transformation Program [4].

The NTP aims at three main goals: achieving governmental operational excellence by raising the quality of services; improving economic enablers by supporting the growth of the private sector, raising labor market attractiveness, ensuring the sustainability of vital resources, and developing the tourism and non-profit sectors; and enhancing living standards with improved systems of social services, health care, and safety. The NTP consists of thirty-seven strategic objectives under eight themes, as shown in figure 1. The health care transformation is one of the eight themes of the NTP [4, 5].

This article aims to describe the new Model of Care (MOC) according to the recent Saudi health care transformation under the King-dom’s vision 2030.

## MATERIALS AND METHODS

Data relating to the Saudi Arabian vision 2030, the national transformation program, the Saudi health system, the transformation of healthcare, and the new MOC, were extracted from the Ministry of Health (MOH) and other relevant websites or portals. A further search of the material and published literature in several databases, including Wikipedia, Google Scholar, PubMed, and Medline, was carried out. We analyzed all the relevant studies, government reports, and documents for their contents, and the information was synthesized and reported.

## RESULTS

### Saudi Health System

The history of health care facilities in the Kingdom is almost a century. The public health department was established first in Mecca in 1925 [6]. After the second world war, the Saudi economy was growing due to the dramatic increase in oil production, and more health care infrastructure was built. In 1950, the MOH was formed with various health care institutions [6]. In the year 2018, Saudi Arabia had 75,225 beds in 484 hospitals, which was 22.5 beds/10,000 population. The total health budget reached 90 billion SR (9.2% of total governmental budget) in 2018 [2].

The Kingdom of Saudi Arabia has made notable progress in improving its population’s health over recent decades, particularly in the areas of child and maternal mortality and the reduction of infectious diseases. Average life expectancy at birth improved from 64 years in 1970 to 75 years in 2016 [7], with new targets set to ensure it increases to 80 years by 2030 [4]. Despite these advances, many health issues still need to be addressed. For example, the rates of avoidable injury and non-communicable disease remain high by regional and international standards. There remains considerable scope to reduce preventable mortality and avoidable morbidity in both the working and elderly populations. All amenable to reduction are areas of concern, including heart disease, stroke, diabetes mellitus, respiratory disease, mental health, road traffic accidents, and congenital diseases [8].

### Transformation of Healthcare

With the growing population, the Saudi health sector faces enormous challenges and is undergoing significant reform following regional and global trends. The First Theme of NTP is “Transform health care”, which aims to restructure the health sector to become a comprehensive and helpful system. A new MOC will promote public health that focuses on the prevention and health awareness of society. It will ensure access to health services through optimal coverage, equitable geographical distribution, and comprehensive & expanded e-health services and digital solutions. Moreover, it will target the continuous improvement of health services by focusing on the beneficiaries’ experience and satisfaction in line with international standards and best practices [4, 5].

Leading organizations involved in health care transformation are the MOH, Saudi Health Council, King Faisal Specialist Hospital and Research Center, Saudi Food and Drug Authority, The Saudi Red Crescent Authority, and the Ministry of Education. Three significant challenges were identified in the health care system: 1) difficult access to health services, 2) limited quality and inefficient health services, and 3) inadequate preventive health care. Different strategies were developed to overcome these challenges. Firstly, to enhance the accessibility of health care services for the citizens, the plan was to expand health facilities, including improving infrastructure and increasing the numbers of beds and health care professionals. Adequate geographical distribution ensures affordable services, easy specialized consultation through workforce planning, redistributing responsibilities, improving referral system and appointments, and easy access to emergency medical care by promoting related medical professions were also planned in this regard. Secondly, to improve the quality and efficiency of health care services, the plan was to increase clinical effectiveness, enhance safety, improving patient experience, and improve sustainability and financial transparency. Finally, regarding promoting prevention against health risks, control communicable and non-communicable diseases, and improve readiness to confront health disasters [4, 5].

### The new Model of Care (MOC)

#### Definition and background

The new MOC theme is a focal point for improved treatment and care modalities individually. There is a global trend of shifting from activity-based to outcome-based payment structures that incentivize better performance and care quality. The health providers are incentivized to manage the population’s health care cost over the long term and help people live healthier lives longer. A shift to increasingly autonomous and Accountable Care Organization (ACOs), delivering care through greater collaboration and integration, and budgetary responsibility are also evident. A National MOC will unlock multiple benefits that are core to the health care Transformation. The main advantages of the MOC, as shown in Table 1 [9].

**Table 1:** The benefits of the new Model of Care (MOC).

Set a blueprint for ACOs to build service provision capabilities and plans on unlocking intrinsic value through integrated services.
Improve patient experience by introducing clear citizen-centric pathways delivering quality, timely and accessible services.
Organizes large scale transformation by regulating and directing a multitude of initiatives towards a common goal.
Enables value-based financing of health care by linking payment mechanisms to MOC pathways and outcomes.
Facilitates national knowledge and capability sharing, patient flow between ACOs, and economies of scale efficiency generation.

ACOs – Accountable Care Organizations.

The MOC concept started with understanding the current state and collecting learnings. The global health care developments and key directional trends also inspired this. More than 60,000 citizens participated in the public survey around the patient-centric design, 2500+ health care professionals engaged in e-discussions, and 1000+ health care professionals surveyed to identify improvement opportunities. The MOC answers six questions from the people’s perspective:

1. How will the health system support the people to keep them well?
2. How will it help during an urgent problem?
3. How will it support them to have an excellent outcome for any planned procedure?
4. How will it help to deliver a healthy baby safely?
5. How will it support during the chronic health conditions?
6. How will it provide them compassionate care during the last phase of life?

**Figure 2:**
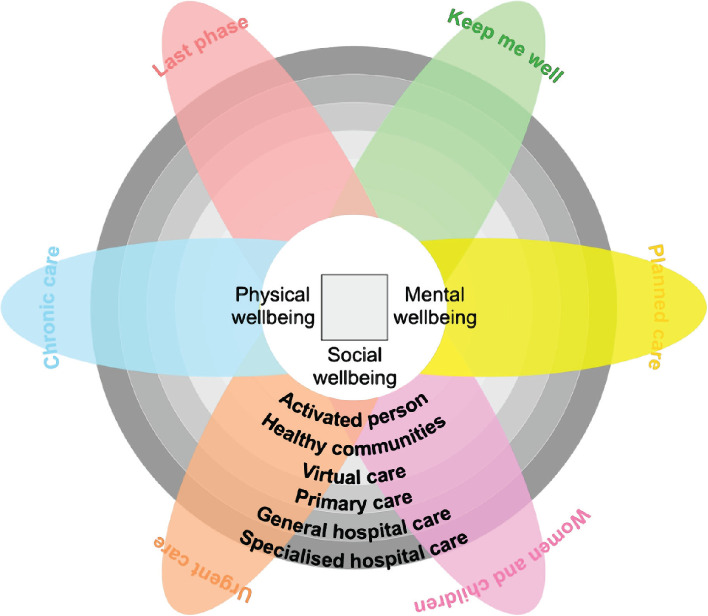
Model of Care: the “Six Systems of Care” and “Service Layers”.

These questions resemble the MOC’s Six Systems of Care (SOC). The SOC is the configuration/set-up of all available services to a patient to address a need: Keeping well, planned procedure, women & children, urgent problems, chronic conditions, and the last phase of life. Again, the SOC is cut across different “service layers” to support people’s stay well and efficiently get them well, too, when they need care (Figure 2) [9].

Activated people are at the core of the MOC. It emphasizes the role that individuals and their families will play in keeping well and taking care of their health through self-care, awareness, and empowerment. Healthy communities will support activated people by encouraging them to lead healthy lifestyles, providing them with the appropriate information, and providing them with access to community care and wellness facilities. Virtual care will be an authoritative source of health advice. In most instances, virtual care will serve as people’s first point of contact with medical care providers, improving people’s access to medical information and guiding them to navigate the health care system and seek appropriate care. Primary care, secondary care, and tertiary and quaternary care will still be the primary source of care beyond virtual care [9].

Although the MOC describes a comprehensive care system for meeting the health needs, implementing it will require six key enablers. These include workforce, eHealth, corporatization, governance, health care financing, and private sector participation.

The Vision Realization Office (VRO) at MOH has primarily been tasked with ensuring the successful execution, monitoring, and evaluation of health care transformation initiatives, as shown below, of which the MOC brings all these initiatives together. The MOC mainly describes a comprehensive care system for meeting health needs and implementing it will require support from six key enablers, shown in Table 2 [9].

**Table 2:** The six key enablers and initiatives by The Vision Realization Office (VRO).

Key enablers	Initiatives
**Private Sector** **Participation**	• Increase private involvement by facilitating ownership, or management of MOH hospitals; • Actively support localization of pharmaceutical and medical devices leveraging MOH procurement.
**e-Health**	• Provide digital tools (apps) for patient self-services, prevention, connected care, and workforce efficiency; • Accelerate IT infrastructure build-up at MOH to reach 100% deployment by 2020.
**Workforce**	• Enhance the quality and quantity of workforce through increased capacity, improved licensing criteria, and making profession attractive; • Establish a National Health care Workforce Planning Unit to coordinate actions across key stakeholders.
**Health care Financing**	• Establish a value-based provider payment system; • Set up National Health Insurance with a gradual rollout.
**Corporatization**	• Split MOH to corporatize delivery, creating independent provider networks with operational autonomy; • Create local clusters that bring providers together, ultimately forming accountable care organizations.
**Governance**	• Strengthen MOH mandate to lead sector reform with strong oversight over regulatory agencies (“super-regulator”) and transform the role of MOH to be more strategic; • Create a range of new development and regulatory bodies at arm’s length from the MOH.

MOH – Ministry of Health

### How was the MOC designed and developed?

The first phase of the MOC project focused on understanding the current situation, designing the new MOC, and defining interventions required for the new national MOC for the Kingdom. From October 2016 to April 2017, the MOH and the VRO led a national effort to transform the health care sector across the Kingdom. Three national workshops (three care design groups; the CDG) were held with the key stakeholders’ participation, including over 450 Saudi doctors, nurses, pharmacists, dentists, and patients (plus an additional 2000 involved in virtual discussions). They worked together to design a comprehensive care system for meeting health needs throughout the Kingdom. In the first workshop (the CDG 1), the participants from different regions and different health care sectors in KSA came together to agree on the key issues facing the current health care services and critical areas of improvement. Based on the key issues and priorities developed in CDG 1, the second workshop participants in the CDG 2 designed and suggested the initial list of interventions for each of the six Systems of Care (SOC) included as part of the new MOC. In the third workshop, the CDG 3, the National SOC Leaders, and international experts, along with other participants, finalized the new MOC design for the Kingdom. It included detailing each of the six SOC’s design that constitutes the MOC and considering how the MOC would be adjusted to serve in different contexts, including the City, Town, Rural, Hajj & Umrah, Mental Health, Children’s needs. His Excellency, the Minister of Health, launched the new MOC on 23 April 2017 [9].

**Figure 3:**
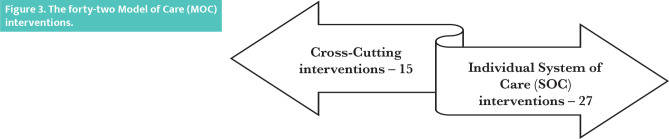
The forty-two Model of Care (MOC) interventions.

**Figure 4:**
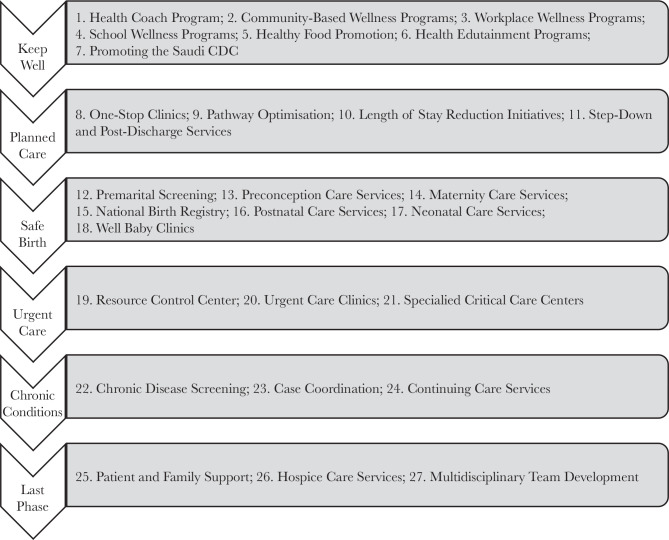
The Twenty-seven Individual SOC interventions.

**Figure 5:**
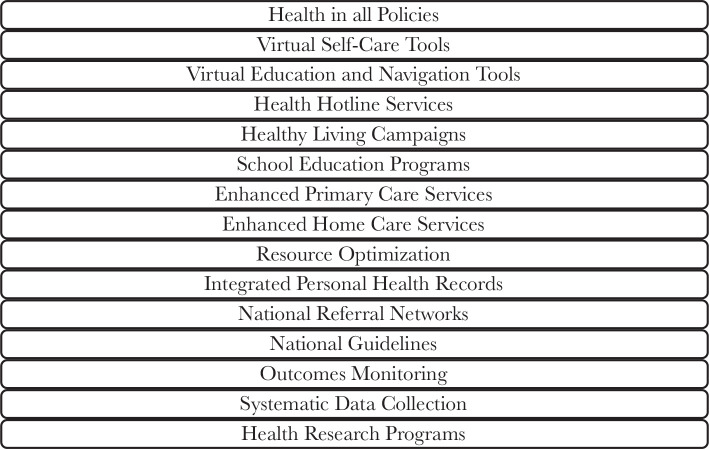
The fifteen cross-cutting interventions.

The new MOC describes a total of forty-two interventions (Figure 3), of which twenty-seven split across the six SOC (Figure 4) and the rest fifteen cut-across the multiple SOC (Figure 5). An intervention is a combination of program strategies designed to produce behavior changes, improve individuals’ health or the population, and/or reduce the cost. Each cross-cutting intervention may be applied to the SOC’s two or more and need to be developed further without isolation within a single SOC [9].

**Figure 6:**
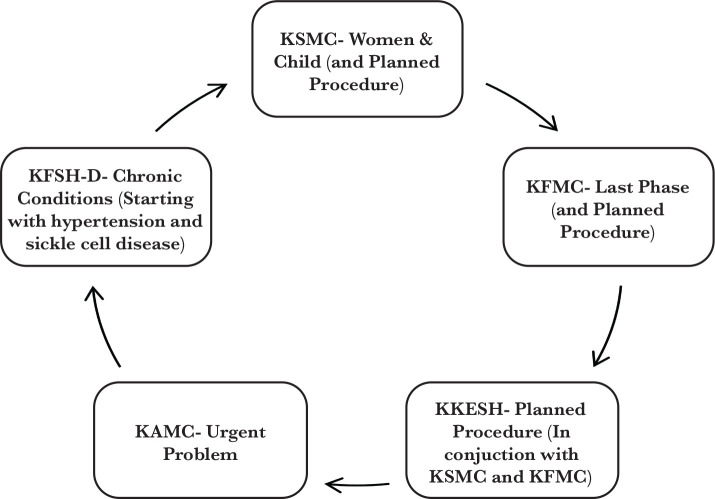
Pathways that will be implemented by each pathfinder.

The second phase of the New MOC was conducted in two parallel workstreams over five months (April to August 2017). The first workstream was the regional pathway development focused on working with five pathfinders to develop the national SOC designs into implementable regional pathways. The pathways are the route that a patient undertakes when accessing the health care system based on their need – this is operationally specific in terms of which provider the patient accesses along this route. The pathfinders refer to the selected Medical Cities (and their surrounding providers) and other hospitals, which have been identified as the first in the country to make the journey towards Accountable Care Organizations (ACOs) and piloting elements of the new MOC. They are King Saud Medical City- Riyadh (KSMC), King Fahad Medical City- Riyadh (KFMC), King Khalid Eye Specialist Hospital- Riyadh (KKESH), King Abdullah Medical City- Mecca (KAMC), and King Fahad Specialist Hospital- Dammam (KFSH-D) (Figure 6) [9].

The second workstream was the national implementation planning focused on working with the national taskforces of experts on designing and planning for the national development of six cross-cutting and “Keep Well” interventions that require standardized national implementation. Based on phase two’s approach and planning outcome, the next phase will implement MOC priority solutions. The “100-day plan” was executed, and the high-level implementation plan was detailed out further. The implementation is likely to occur in waves based on clusters. A cluster refers to a group of health care providers who will form an ACO in the future. They are geographically defined around a Medical City or other large hospital as a hub. It is estimated that there will be 20–30 clusters across the Kingdom. Each health care provider (including hospitals, Primary Health Clinics etc.) within a cluster will be required to coordinate and collaborate to meet a defined population’s needs. Each cluster may eventually have a set budget allocation and work under a contract that specifies the outcomes and other objectives required to achieve within that budget [9].

## DISCUSSION

The MOH has struggled to build the inter-ministerial dialogue needed to address some pressing issues. Similarly, other government agencies in the Kingdom do not often consider the health and healthcare ramifications of their decisions while developing major policy initiatives. Over the next decade, significant problems need to be addressed to modernize the Saudi healthcare system and fulfill the “Vision 2030”.

The Kingdom’s population continues to rise and is expected to be 39.5 million, including 4.63 million elderly (60–79 years) by 2030. One-third of the Kingdom’s population is expatriate (10 million in 2015), primarily adult workers. Many international visitors, particularly during the major religious festivals (Hajj and Umrah), have an additional health burden on the Saudi health system. In recent years approximately 1.8 million foreign pilgrims arrived, and the overall number of foreign pilgrims visiting Mecca is estimated to be as high as three million in some years [10]. The people living in urban areas are expected to rise from 83.3% (2016) to 85.9% by 2030. The rates of avoidable injury and non-communicable disease in the Kingdom remain high compared to regional and international standards. We must improve non-communicable illness and accident prevention to reduce avoidable illness and death. Significant outbreaks of infectious diseases are still possible, especially during Hajj or in the aftermath of natural or human-made disasters.

Primary healthcare continues to be insufficient and inconsistent in the Kingdom. The Secondary, tertiary, or specialized hospitals and related services are dispersed throughout the Kingdom. The rehabilitation, long-term, and home care services are insufficient throughout the country. There are significant inconsistencies in the quality of patient care. Most of this is due to a lack of standardized treatment plans and pathways and insufficient monitoring of patient processes and outcomes. In 2015, The Saudi Central Board for Accreditation of Healthcare Institutions (CBAHI) found these deficiencies during the Essential Safety Requirements Survey in hospitals of all types [9].

The population measures provision, access, and expenditure served rather than the patients treated; there is unjustified variance. It included both overuse and underuse, resulting in substantial value and efficiency shortfalls. Rather than being patient or individual-centric, the system is now resource and staff-centric. It’s also focused on institutions rather than people. A health system must be both open and attentive to the overall well-being of patients. There are significant capacity and skill gaps in the workforce, especially among Saudi employees. Additionally, the health system lacks robust, consistent, and integrated digital information systems across all hospitals. It may help quantify and manage resources, activity levels, product quality, and performance [9].

The New MOC, provider reforms, financing reforms, governance growth, private and third sector engagement, workforce development, and eHealth development are the seven themes that the VRO has focused on for working around. The first three themes can be viewed as enablers of three distinct degrees of worth. The MOC theme is a focal point for enhancing personal value by improving treatment and care modalities at an individual level. The provider theme is a focal point for enhancing utilization value at an intermediate level, whether at the clinical micro-system, hospital, or local health system level. The financing theme serves as a focal point for improving allocative performance by ensuring that intermediate levels receive optimal resource levels based on patients’ needs and capacity to benefit. Financing, it could be argued, plays a direct role in ensuring all three forms of value [9].

Furthermore, patients’ needs are often qualified by other factors such as the patients’ merits, economic goals to preserve the working population’s health, or the patients’ capacity and willingness to pay. Previous experience with health transformation initiatives indicates that organizational and financial reforms are unlikely to result in significant performance improvements unless followed by supply-side enhancements, such as increased productivity, efficacy, equity, and public health responsiveness. It’s essential to understand how the three value dimensions are interconnected and reinforce each other. Completing all seven work themes would be critical to our transition strategy’s overall success [11].

## CONCLUSION

A longer timeframe and additional resources will require to roll out all – the MOC solutions and the enabler workstreams’ local implementations. Implementation of all MOC interventions will streamline the Saudi health care system to embrace the Kingdom’s “Vision 2030”. Additionally, the health sector would reduce government spending and the diversification of the Saudi economy. It is essential to address the possibility of long-term drops in crude oil prices and the effects on government revenues. As a result, the Kingdom must encourage intervention both inside and outside the health system to minimize accidents and the primary and secondary prevention of non-communicable diseases. Also, implement systematic assessments of population needs and health system efficiency to optimize resource distribution and provide the results that people require.

## References

[1] Walston S, Al-Harbi Y, Al-Omar B (2008). The changing face of health care in Saudi Arabia. Ann Saudi Med.

[2] MOH Statistical Yearbook 2018 Kingdom of Saudi Arabia - Ministry of Health Portal. https://www.MOH.gov.sa/en/Ministry/Statistics/book/Documents/book-Statistics.pdf.

[3] Economy of Saudi Arabia Wikipedia. https://en.wikipedia.org/wiki/Economy_of_Saudi_Arabia#cite_note-13.

[4] National Transformation Program (2030). Saudi Vision. https://vision2030.gov.sa/sites/default/files/attachments/NTP%20English%20Public%20Document_2810.pdf.

[5] (2019). Saudi Vision 2030. https://vision2030.gov.sa/sites/default/files/report/Saudi_Vision2030_EN_2017.pdf.

[6] Al-Hanawi MK, Khan SA, Al-Borie HM (2019). Health care human resource development in Saudi Arabia: emerging challenges and opportunities–a critical review. Public Health Rev.

[7] WHO Saudi Arabia Country File. World Health Organization.

[8] WHO (2014). Global status report on non-communicable diseases 2014. World Health Organization.

[9] MOH (2019). Health Sector Transformation Strategy V.3. https://www.moh.gov.sa/en/Ministry/vro/Documents/Healthcare-Transformation-Strategy.pdf.

[10] Hajj Wikipedia. https://en.wikipedia.org/wiki/Hajj#Number_of_pilgrims_per_year.

[11] Saltman RB, Bankauskaite V (2006). Conceptualizing decentralization in European health systems: a functional perspective. Health Econ Policy Law.

